# Influenza and Insanity

**Published:** 1897-03-13

**Authors:** 


### The Hospital

A Journal of

The Medical Sciences and Hospital Administration.

Vol. XXI.—No. 546.] [March 13, 1897.

### Influenza and Insanity.

Few medical men who have had considerable opportunities of studying influenza in its remoter as well as in its more immediate sequelæ will be surprised to read the verdict given by Dr. T. S. Clouston, Medical Superintendent of the Royal Edinburgh Asylum for the Insane, concerning the abiding and serious effects of that strange disease upon the nervous system. "Probably no such destroyer of nervous energy," says Dr. Clouston, "and no such producer of nervous disease, as the influenza poison, has appeared in the world in recent times." To him, as a specialist in nervous disorders, it is "the most striking medical fact of his time."

Mania and melancholia, undue exaltation and undue depression, are two types of mental disease which stand to each other in the relation of direct contrast. Under certain sets of conditions mania will be the predominating type; under certain other sets, melancholia. Conditions which profoundly depress the nervous system culminate in the melancholic type of insanity. All this is familiar, not to the alienist only, but to the general practitioner of experience. Certain facts, to which Dr. Clouston gives prominence, bring out in a very striking way the profoundly depressing effects of influenza upon the nervous system, effects which continue for years, and may only cease with life. In the seven years beginning with 1883, and ending with 1890, the cases of mania at the Morningside Asylum far outnumbered those of melancholia. On an average there were 45 more cases in each year. That is a common experience at lunatic asylums. But during the seven years period just closed, that is 1890-97, the average of mania cases has only been 18 more than that of melancholia. The most striking fact of all which Dr. Clouston publishes is this: that in the three years 1890-91-92, the years of the greatest prevalence of influenza, the cases of melancholia actually outnumbered those of mania, and that during the whole of the three years. To influenza, and to influenza alone, Dr. Clouston attributes these striking variations in the usual circumstances of the history of insanity.

Practical commentaries upon facts of this kind do not at once suggest themselves. There are, however, two or three which seem to be of importance. The first is that if influenza, besides being so deadly to life, is justly to be credited with the worse than deadly consequences of melancholic insanity, a little more energy ought to be displayed in the direction of prevention. If Dr. Clouston's verdict be right, it will not be easy to mention a more potent enemy of civilisation and human happiness than this destructive disease. The next suggestion is one for treatment. Undoubtedly of all the general principles of treatment those which are included under stimulation and judicious feeding are the most important. If the depressing influences once make headway; if they are allowed to operate unchecked for even a very few days, not only may a slow recovery be expected, but permanent eff[illegible]ts of the kind mentioned by Dr. Clouston, a[illegible]d other injurious sequelæ, which every professional reader can recall for himself, will almost certa[illegible]nly result. At the very first outset of an attack of influenza steps should be taken to husband the strength and supply abundance of nourishing food. If these principles are borne in mind, and carried out with intelligence and unflinching perseverance, convalescence will be more rapid, and the immediate and more remote sequelæ will be much diminished in severity and duration.

It may seem to some that these convictions are given expression to a "little out of due time." That, however, is by no means so. There has been a considerable prevalence of influenza during the past few months, and there have been atmospheric and other weather conditions which have greatly favoured its spread. The season is by no means so far advanced but that the disease might yet assume epidemic proportions; and if it should, it is pretty certain that those who are now experiencing a low average of health and vigour will suffer greatly.

But, indeed, no apology need be made for bringing into prominence the fact of the deadliness of such a disease as influenza, and of its extraordinary power to do worse than kill—to drive mad. As Dr. Clouston reminds the profession, medicine is no longer a mere art of healing, its "highest aim," he tells us, is the aim which it has in common with hygiene and education, the great aim of "making man's mind larger and sounder." The last penalty of Nature, the penalty which she inflicts with absolute impartiality on all who willingly or unwillingly infringe her laws, is that of a physiological condition which ends in one or other of the varieties of physiological dissolution. The terrors which physiology holds out to the unwise and the disobedient are not the doubtful terrors of the world to come, but the certain and altogether unavoidable terrors of the world that now is; the terrors of broken health, and the still more dreadful horrors of a ruined mind.

In a strenuous period like the present the sound mind is our one salvation. No intelligence is wasted which devotes itself on the large scale to the preservation of the mind of the nation. No warnings can be too terrible which have for their object the scaring off of hard-worked and much-suffering men and women from the quicksands of insanity. Many a time and often is the practising physician at the centre of a large population asked the melancholy question, "Is life worth living?" If life be so spent that insanity is and must be its almost certain final stage, there is one answer, and only one to this question—"Life is not worth living."

